# Diagnostic delay and mortality of active tuberculosis in patients after kidney transplantation in a tertiary care hospital in China

**DOI:** 10.1371/journal.pone.0195695

**Published:** 2018-04-16

**Authors:** Wei Wu, Meifang Yang, Min Xu, Cheng Ding, Yongtao Li, Kaijin Xu, Jifang Shen, Lanjuan Li

**Affiliations:** 1 State Key Laboratory for Diagnosis and Treatment of Infectious Disease, The First Affiliated Hospital, School of Medicine, Zhejiang University, Hangzhou, Zhejiang, People’s Republic of China; 2 Collaborative Innovation Center for Diagnosis and Treatment of Infectious Diseases, Hangzhou, Zhejiang, People’s Republic of China; Chinese Academy of Medical Sciences and Peking Union Medical College, CHINA

## Abstract

TB infection in patients after kidney transplantation remains a concern in a successful long-term outcome. This retrospective, descriptive study was performed on tuberculosis infection after kidney transplantation in the Department of Infectious Disease of the First Affiliated Hospital of Zhejiang University, a tertiary care hospital in China, from January 2011 to April 2017, with the aim to explain the clinical features of active tuberculosis after kidney transplantation and explore the correlated factors for diagnostic delay and mortality. It included 48 cases. All these cases were followed up for at least 12 months after anti-tuberculosis therapy, except the ones who died during this period. The median time of transplantation to active tuberculosis of these 48 patients was about 5.4 years. The time from a first hospital visit to the diagnosis (diagnostic delay) of 12 (25%) cases was more than 30 days. The correlated factors for the diagnostic delay more than 30 days were a fever for more than 2 weeks and antibiotic use for more than 2 weeks. Nine (18.8%) cases died during the anti-tuberculosis therapy or following-up period due to TB relapse. The risk factors for mortality were severe complications, such as encephaledema, severe pneumonia, intestinal perforation, liver function failure, and the following multiple-organ failure. In conclusion, the possibility of tuberculosis infection should be carefully assessed and sometimes diagnostic anti-tuberculosis therapy may be required for patients who had a fever for more than 2 weeks or used antibiotics for more than 2 weeks after kidney transplantation. Severe complications and the following multiple-organ failure might increase the mortality among these patients.

## Introduction

Transplantation (TPL) is a therapeutic option for end-stage organ disease. Recipients receive immunosuppressive agents after TPL to prevent rejection, which causes impairment of their immune state and thus results in an increased risk of infectious complications [1]. Post-transplant tuberculosis (TB) is a constraint in successful long-term outcomes of organ transplant recipients and a life-threatening infection. Among solid-organ transplant patients, an estimated frequency of active TB disease is found to be 20–74 times that of the general population. But the frequency differs according to the organ transplanted. The incidence of active TB after solid-organ transplant is about 1.52%–2.29% in China [2].

The number of studies available on TB infection after solid-organ transplantation has increased rapidly in recent years. Most of these studies were focused on the incidence or epidemiology of post-transplantation patients [3–5] or the clinical characteristics and outcomes compared with the general population[6–8]. These studies also highlighted several challenges in diagnosing and treating TB in organ transplant recipients[9]. First, since these patients may be affected by immunosuppressive agents after operation, they may have atypical clinical presentation, negative or indeterminate tuberculin skin tests (TST) or interferon-gamma release assays, and negative sputum smear or culture results. Impediments to rapid and accurate diagnosis may lead to delay in diagnosis and treatment. The literature has reported increased mortality, clinical severity and transmission of disease in the cases of treatment delay in immunocompetent patients with TB [10–11], but a few were discussed in post-transplantation patients. Second, therapeutic challenges arise from drug-related toxicities and metabolic interactions between immunosuppressive and anti-TB drugs in post-transplantation patients [12]. The crude mortality of post-transplant TB was about 20%–30%[9]. Some case reports showed deaths of these patients having TB-related complications or co-infections. However a few explored the risk factors for mortality of TB infection after kidney transplantation.

The present study retrospectively analyzed the clinical data of patients with active TB infection after kidney transplantation in First Affiliated Hospital of Zhejiang University. It also explored the correlated factors for diagnostic delay and mortality, with the over-arching objective of improving the early diagnosis and survival of these patients.

## Materials and methods

### Setting and patients

We searched the database of kidney transplantation for patients who were diagnosed as “active tuberculosis” for the first time from January 2011 to April 2016 in the First Affiliated Hospital of Zhejiang University, a tertiary care hospital in China. All these patients had received kidney transplantation in this hospital. None of the transplant donors in this study were from a vulnerable population and all donors or next of kin provided written informed consent that was freely given.

Totally 53 patients were included in this study from January to April 2016 but 5 of them were proved to be nontuberculous mycobacteria (NTM) in the following up period by culture results. So totally 48 participants were recruited. 47 of these patients had received anti-tuberculosis therapy and one case was diagnosed after death without therapy. All of these patients were followed up for at least one year after anti-TB therapy till April 2017, monthly during the treatment time and quarterly during the following up period, except the ones who died during this period. The demographic and clinical data such as patient characteristics, clinical–epidemiological profile, diagnosis, and outcome of these patients were collected from January 2016 to April 2017. Doctors from the departments of infectious disease and kidney transplantation analyzed the data together. Two senior radiologists conducted computed tomography (CT) or magnetic resonance imaging (MRI), and two senior pathologists diagnosed the biopsy samples. The ethics committee of the First Affiliated Hospital, College of Medicine, Zhejiang University approved this study. All participants or their relatives signed the informed consent form before the study. The data regarding age, gender, or occupation were revealed with patients’ consent. The authors had access to information that could identify individual participants during or after data collection.

### Criteria for active TB diagnosis, laboratory tests, and immunosuppressant treatment

The active TB case definition was based on the World Health Organization criteria[13]: (1) identification of the bacilli in a biological specimen by smear microscopy and/or culture or (2) a clinically diagnosed case without bacteriological confirmation when suggestive imaging or biopsy results were sufficient for a specialist to indicate treatment. The gold standard diagnosis of TB was made when *Mycobacterium tuberculosis* (MTB) was isolated.

Laboratory tests for TB infection included acid-fast bacilli smear or culture, T-spot TB, and GeneXpert. Acid-fast bacilli culture was performed using bact960 (Becton, Dickinson and Company, NJ, USA) from 2004. T-spot TB was performed using an enzyme-linked immunospot assay kit (Oxford Immunotec Ltd, Abingdon, UK) from 2011. The rapid nucleic acid amplification technique GeneXpert MTB/RIF (Cepheid Inc, CA, USA), an automated molecular test for MTB and resistance to rifampicin (RIF), was used from 2013, which could increase the sensitivity and decrease the time to diagnosis.

Tacrolimus (FK506) or cyclosporin A (CsA) with mycophenolate mofetil and glucocorticoid was used after kidney transplantation. High doses of methylprednisolone were used for patients with acute rejection after transplantation.

### Treatment and outcome of active TB

Patients with pulmonary TB (PTB) used a four-drug regimen of isoniazid, rifampin or rifapentine, ethambutol, and pyrazinamide for the first 2 months (intensive phase), followed by isoniazid and rifampin for an additional 4 months (continuation phase). EPTB, miliary TB, disseminated TB, or tuberculosis meningitis needed a longer duration of therapy. Fluoroquinolones, including moxifloxacin and levofloxacin, were used for individuals having poor liver function or hepatotoxicity on standard TB therapy.

The outcome of therapy included treatment completed, relapse, and death. Treatment completed: Patients received a full course of treatment and had not become smear-positive at the end of treatment. Relapse: Patients completed the active TB therapy, but TB relapsed during the follow-up period. Death: Patients died during the course of TB treatment or following up period due to TB-related complications. All the patients were followed up for at least 12 months after anti-TB therapy except the ones who died during this period.

### Screening and treatment of latent TB

Chest xray (CXR) or computed tomography (CT) were essential for screening latent TB infection (LTBI) pre-transplant, and were conducted regularly post-transplant in our center. PPD were used for screening but gradually replaced by T-spot after 2011. T-spot test was recommended in diagnosis of both active TB and latent TB infection in pre- and post-transplant setting. A 9-month INH monotherapy or sometimes alternative drug were empirically used for LTBI since 2014, after the publication of guideline for “Mycobacterium tuberculosis infections in solid organ transplantation” [9].

### Definitions of severe TB and diagnostic delay

PTB[14]: Patients with exclusively intrathoracic involvement (i.e., confined to lung parenchyma, pleura, and intrathoracic lymph nodes) were considered to have PTB. EPTB: Patients with pulmonary involvement, who also had an extension of the disease to organs or tissues outside the thorax, were classified as having EPTB. Disseminated TB (DTB): Patients having ≥2 noncontiguous sites with TB lesions were considered to have disseminated TB. Severe TB[15]: Severe TB included patients with miliary TB, DTB, or tuberculosis meningitis.

When alanine aminotransferase levels were ≥3 times the upper limit of normal in the presence of symptoms or ≥5 times the upper limit of normal in the absence of symptoms, it was established that antituberculous drugs were hepatotoxic.

Bacillus Calmette-Guerin (BCG) vaccinated: Previous BCG vaccination was determined either by the presence of a scar on the left upper arm or record of BCG vaccination. Close contact: One living in the same household or in frequent contact with a source case with sputum smear-positive or culture-positive TB was considered a close contact.

Patient delay: It was defined as the time from the onset of symptoms to the first interaction with health services. Diagnostic delay^:^ It was defined as the time from the first hospital visit to initiation of treatment. Treatment delay: It was defined as the duration of time from the onset of symptoms to the initiation of treatment, including patient delay and diagnostic delay.

## Statistical analysis

All data in this study were managed and analyzed using SPSS 20.0 (SPSS, IL, USA). Continuous variables were presented as mean ± standard deviation, and non-normally distributed variables as median (quartile range). Categorical variables were presented as counts and proportions. The *t* test, chi-square test, Fisher’s exact test, or Mann–Whitney *U* test was used to compare variables between groups. All statistical hypothesis tests were two sided, and P values <0.05 were considered to have statistical significance.

## Results

### 1. Demographic and clinical characteristics of 48 patients with active TB after transplantation (Table 1)

**Table 1:** Demographic and clinical characteristics of 48 patients with active tuberculosis after transplantation.

Characteristic	Total (*n* = 48)
**Age (year)**	45.1 (22–67)
**Gender**	
Male, *n* (%)	39 (81.3)
Female, *n* (%)	9 (18.7)
**Time from TPL to TB (year)**	5.4 (1 month to 18 years)
Within the first year after TPL, *n* (%)	17 (35.4)
**Acute rejection after TPL, *n* (%)**	9 (18.8)
**Comorbidities**	
Hypertension, *n* (%)	29 (60.4)
Viral hepatitis B, *n* (%)	17 (35.4)
Diabetes mellitus, *n* (%)	12 (25)
Viral hepatitis C, *n* (%)	3 (6.3)
**Past TB history**, *n* (%)	2(4.2)
**Contact history of TB at home**, *n* (%)	1 (2.1)
**Patient delay (day)**	24.8 (1––180)
≥30, *n* (%)	14 (29.2)
**Diagnostic delay (days)**	25.9 (1–180)
≥30, *n* (%)	12 (25)
**Primary symptoms during hospital visit**	
Fever, *n* (%)	42 (87.5)
Fever ≥2 weeks, *n* (%)	24 (50)
Cough or expectoration, *n* (%)	22 (45.8)
Dyspnea, *n* (%)	14 (29.2)
Headache, *n* (%)	6 (12.5)
Abdominal pain, *n* (%)	5 (10.4)
Sperficial lymphadenectasis, *n* (%)	5 (10.4)
Frequent or urgent micturition, *n* (%)	3 (6.25)
**Finding of lung CT scan**	
Nodule or speckle, *n* (%)	33 (68.8)
Focal proliferation, *n* (%)	20 (41.7)
Pleural effusion, *n* (%)	15 (31.3)
Hilar or mediastinal lymphadenectasis, *n* (%)	10 (20.8)
Miliary tuberculosis, *n* (%)	8 (16.7)
Cavity, *n* (%)	5 (10.4)
Consolidation, *n* (%)	3 (6.25)
Bilateral pulmonary lobe, *n* (%)	28 (58.3)
Unilateral pulmonary lobe, *n* (%)	16 (33.3)
**Lab test**	
T-spot positive, *n* (%)	28 (58.3)
T-spot negative, *n* (%)	6 (12.5)
T-spot ND, *n* (%)	14 (29.2)
**Severe complications**	
Liver function failure, *n* (%)	4 (8.3)
Encephaledema, *n* (%)	4 (8.3)
Severe pneumonia, *n* (%)	1(2.1)
Intestinal perforation, *n* (%)	1 (2.1)
Pneumonia and intestinal perforation	1(2.1)
**Outcomes**	
Completed, *n* (%)	39 (81.2)
Died, *n* (%)	9 (18.8)

Note: CRP, C-reactive protein; CT, computed tomography; ESR, erythrocyte sedimentation rate; ND, not done; TB, tuberculosis; TPL: transplantation.

The average age of onset of 48 cases of active TB after kidney transplantation was 45.1 (22–67) years. Of these 39 (81.3%) cases were male, and 9 (18.8%) cases had acute rejection after operations. Only two persons had a definite history of active TB, and one person had a contact history of TB at home. The average time from transplantation to TB was 5.4 years (1 month to 18 years). The average time from the onset of symptoms to hospital visit (patient delay) was 24.7 days (1–180 days); in 14 cases (29.2%), it was longer than 30 days. The average time from the hospital visit to diagnosis (diagnosis delay) was 25.9 days (1–180 days); in 12 (25%) cases, it was longer than 30 days. Further, 42 (87.5%) patients had a fever during the first hospital visit, whereas 24 (50%) had a fever for ≥2 weeks.

CT of lung showed the following results: 33 (68.8%) of nodule or speckle, 20 (41.7%) of focal proliferation, which may be caused by chronic granulomatous inflammation of TB infection. 15 (31.3%) of pleural effusion, 10 (20.8%) of hilar or mediastinal lymphadenectasis, 8 (16.7%) cases of miliary TB, 5 (10.4%) of cavity, 3 (6.25%) of consolidation. 28 (58.3%) of bilateral pulmonary lobe, and 16 (33.3%) of unilateral pulmonary lobe. The lab test showed that 28 (58.3%) cases were T-spot positive, 6 (12.5%) were T-spot negative, 14 (29.2%) did not undergo the T-spot test.

Complications and outcomes: A total of 39 (81.2%) cases completed the TB therapy and improved. 9(18.8%) patients died, seven patients died during first anti-TB therapy, and two died due to TB relapse with encephaledema during their follow-up visit. Further, 11(22.9%) cases had severe complications during anti-TB therapy, 4(8.3%) had liver function failure, 4(8.3%) had encephaledema, one had severe pneumonia, one had intestinal perforation, and one case had both severe pneumonia and intestinal perforation.

### 2. Clinical diagnosis of 48 patients with active tuberculosis after transplantation (Table 2)

**Table 2:** Clinical diagnosis of 48 patients with active tuberculosis after transplantations.

Diagnosis	Total (*n* = 48)
**Nonsevere TB**, *n* (%)	29 (60.4)
Pulmonary TB, *n* (%)	24(50)
With pleural TB, *n* (%)	3 (6.3)
With contiguous lymphatic TB, *n* (%)	2 (4.2)
Pleural TB, *n* (%)	3 (6.3)
Lymphatic TB, *n* (%)	1 (2.1)
Urinary tract TB, *n* (%)	1 (2.1)
**Severe TB**, *n* (%)	19 (39.6)
Pulmonary and intestinal TB, *n* (%)	4 (8.3)
Pericardium TB, *n* (%)	2 (4.2)
With pulmonary TB, *n* (%)	1 (2.1)
With pleural TB, *n* (%)	1 (2.1)
Tuberculous meningitis, *n* (%)	5 (10.4)
With abdomen wall and skin TB, *n* (%)	1 (2.1)
With pulmonary TB, *n* (%)	1 (2.1)
Miliary TB, *n* (%)	8 (16.7)
With intestinal TB, *n* (%)	1 (2.1)
With lumbar spine and meningitis TB, *n* (%)	1 (2.1)
With urinary tract TB, *n* (%)	1 (2.1)
With skin TB, *n* (%)	1 (2.1)
With liver TB, *n* (%)	1 (2.1)
**Proof for diagnosis**	
Bacteriological confirmation, *n* (%)	26 (54.2)
Sputum smear or culture, *n* (%)	14 (29.2)
Secretion smear, *n* (%)	4 (8.3)
Urinary sediment smear, *n* (%)	1 (2.1)
GenExpert positive in CSF, *n* (%)	1 (2.1)
Tissue of biopsy, *n* (%)	6 (12.5)
Clinical diagnosis, *n* (%)	22 (45.8)
Granulomatous inflammation and necrosis in biopsy, *n* (%)	4 (8.3)

Note: CSF, Cerebrospinal fluid; TB, tuberculosis.

29 (60.4%) cases were diagnosed with nonsevere TB and the other 19 (39.6%) cases had severe TB. Among the severe TB, four (8.3%) cases had pulmonary and intestinal TB. Two (4.2%) cases were pericardium TB with pulmonary or pleural TB. Five (10.4%) cases had tuberculous meningitis, and eight (16.7%) cases had miliary TB.

26 (54.2%) cases were diagnosed with bacteriological confirmation. Of these, 14 (29.2%) cases were MTB positive in sputum smear or culture,4 (8.3%) cases were positive in secretion smear, one (2.1%) was positive in urinary sediment smear, 6 (12.5%) were positive in tissue of biopsy. And one (2.1%) was MTB positive in cerebrospinal fluid (CSF) with GeneXpert MTB/RIF. The other 22 (45.8%) cases were clinically diagnosed without bacteriological confirmation.

### 3. Diagnostic delay and outcomes of 48 patients with active TB after transplantation

Patients who had a fever for more than 2 weeks (*P* = 0.03) and who had used antibiotics for more than 2 weeks (*P* < 0.001) were significantly associated with diagnostic delay more than 30 days (Table 3). No significant relationship was found for diagnostic delay and severe TB (*P* > 0.05), and diagnostic delay and death (*P*>0.05).

**Table 3:** Related factors for diagnostic delay (≥30 days).

	Diagnostic delay <30 days (*n* = 36)	Diagnostic delay ≥30 days (*n* = 12)	Statistics	*P* value
**Gender**				
Male:Female	27:9	12:0	2.234	0.135^c^
**Age—(year)**	44.11 ± 12.29	48 ± 10.89	-0.975	0.335^a^
**Time from TPL to TB (year)**	2.71 (0.77–6.75)	9.50 (0.88–11.50)	165.5	0.229^e^
**Acute rejection after TPL**, *n* (%)	6 (16.7)	3 (25.0)	0.046	0.831^c^
**Comorbidities**				
Hypertension, *n* (%)	21 (58.3)	8 (66.7)	0.029	0.865^c^
Diabetes mellitus, *n* (%)	11 (30.6)	1 (8.3)	1.333	0.248^c^
Viral hepatitis B, *n* (%)	11 (30.6)	6 (50)	0.759	0.384^c^
Viral hepatitis C, *n* (%)	3 (8.3)	0 (0)	–	0.563^d^
**Patient delay ≥30 days**, *n* (%)	9 (25)	5 (41.7)	0.538	0.463^c^
**Fever ≥2 weeks: fever total**	14:30	10:12	4.706	0.030^b^
**Bilateral pulmonary lobe**, *n* (%)	21 (58.3)	7 (58.3)	0.029	1.000^b^
**T-spot positive:negative**	23:4	5:2	–	0.580^d^
**Bacteriological confirmation**, *n* (%)	18 (50.0)	8 (66.7)	1.007	0.316^b^
**Severe TB**, *n* (%)	12 (33.3)	7(58.3)	1.423	0.233^c^
**Death**, *n* (%)	5 (13.9)	4 (33.3)	1.14	0.286^c^
**Antibiotic use ≥2 weeks**, *n* (%)	2 (5.6)	11 (91.7)	29.574	<0.001^c^

Note:

^a,^ *t* test;

^b,^ χ^2^ test;

^c,^ continuity-corrected χ^2^ test;

^d,^ Fisher’s exact test;

^e,^ Mann–Whitney *U* test.

TB, Tuberculosis; TPL, transplantation.

Severe complications during or after anti-TB therapy due to TB relapse, including encephaledema, severe pneumonia, intestinal perforation, and liver function failure (*P* = 0.017, *P* = 0.032, *P* = 0.032, and *P* = 0.017, respectively), and the sequential multiple-organ failure (*P*< 0.001) were found to have significant association with treatment outcome of death (Table 4). No significant difference was found for severe TB and death (*P* > 0.05).

**Table 4:** Risk factors for death during anti-tuberculosis therapy.

	Completed (*n* = 39)	Died (*n* = 9)	Statistics	*P* value
**Gender**				
Male:Female	33:6	6:3	0.593	0.441^c^
**Age (year)**	44.77 ± 11.39	46.44 ± 14.88	–0.375	0.709^a^
**Time from TPL to TB (year)**	3.00 (0.58–8.00)	10.00 (0.88–13.50)	126.5	0.195^e^
**Acute rejection after TPL**, *n* (%)	6 (15.4)	3 (3.3)	0.593	0.441^c^
**Comorbidities**				
Hypertension, *n* (%)	25 (64.1)	4 (44.4)	0.503	0.478^c^
Diabetes mellitus, *n* (%)	8 (20.5)	4 (44.4)	1.14	0.286^c^
Viral hepatitis B, *n* (%)	12 (30.8)	5 (55.6)	1.03	0.310^c^
Viral hepatitis C, *n* (%)	2 (5.1)	1 (11.1)	–	0.472^d^
**Patient delay**≥**30d**, *n* (%)	10 (25.6)	4 (44.4)	0.507	0.477^c^
**Diagnostic delay**≥**30 days**, *n* (%)	8 (20.5)	4 (44.4)	1.14	0.286^c^
**Severe TB**, *n* (%)	15 (38.5)	4 (44.4)		1.000^c^
**Liver function disorder duration therapy**, *n* (%)	9 (23.1)	3 (50)	0.046	0.831^c^
**Therapy strategy (intensive phase)**				
HR(L)ZE, *n* (%)	4 (10.3)	2 (22.2)		
HR(L)Emfx(Lfx), *n* (%)	30 (76.9)	5 (55.6)	1.702	0.558^d^
HR(L)E, *n* (%)	5 (12.8)	1 (11.1)		
**FK506:CsA**	29:10	8:1	0.245	0.621^c^
**Multiple-organ failure**, *n* (%)	0	9 (100)	41.66	<0.001^c^
**Severe complications**	2 (5.1)	9 (100)	32.082	<0.001^c^
Encephaledema, *n* (%)	1 (2.6)	3 (33.3)	–	0.017^d^
Severe pneumonia, *n* (%)	0	2 (22.2)	–	0.032^d^
Intestinal perforation, *n* (%)	0	2 (22.2)	–	0.032^d^
Liver function failure, *n* (%)	1 (2.6)	3 (33.3)	–	0.017^d^

Note:

^a,^ *t* test;

^b,^ χ^2^ test;

^c,^ continuity-corrected χ^2^ test;

^d,^ Fisher’s exact test;

^e,^ Mann–Whitney *U* test;

CsA, Cyclosporin A; E, ethambutol; FK506, tacrolimus; H, isoniazid; Lfx, levofloxacin; L, Rifapentine; Mfx, moxifloxacin; R, rifampicin; TB, tuberculosis; TPL, transplantation; Z, Pyrazinamide.

### 4. Antibiotic use among 48 patients with active TB after transplantation

Eleven cases used oral or injection antibiotics for more than 2 weeks among the 12 cases with diagnostic delay more than 30 days. Of these, four cases used quinolone antibiotics such as Moxifloxacin and Ofloxacin, which were also recommended for treating rifampicin-resistant and multidrug-resistant TB [16]. Carbapenems were used in four cases; they were the add-on agents for multidrug-resistant TB. Linezolid was used in combination in one case; it was used as the other core second-line agent for multidrug-resistant TB. Compound penicillin or cephalosporin was used in six cases alone or in combination.

## Discussion

The first case of allograft kidney transplantation was performed in July 1977 in the First Affiliated Hospital of School of Medicine, Zhejiang University, which now becomes one of the main transplantation centers in China. However, TB infection in patients after kidney transplantation remains a concern in a successful long-term outcome. In this study, 48 cases of active TB post-transplantation were diagnosed and followed up in the aforementioned hospital from January 2011 to April 2017. The clinical data of patients and the correlated factors for diagnostic delay and mortality were analyzed to improve the early diagnosis and survival of these patients.

TB infection after transplantation is thought to occur due to the reaction of old foci of TB infection. It may also be transmitted from the donor through transplantation [17–18]. Only a small number of cases have been reported to have the primary TB infection post-transplantation, with the rate of primary infection likely greater in developing countries. More than two thirds of reported cases of active TB disease in transplant recipients occurred in the first post-transplant year with the median time of 6–11 months, as shown in a previous report [19]. However, our present study showed that 17 (35.4%) cases had active TB in the first year with the median time of 5.4 years. Only two cases in the present study had a history of active TB infection, and one case had a history of close TB contact. Most patients could not provide a definite history of TB infection, close contact, or TST results before transplantation. None of them had received prophylaxis therapy for latent TB pre-transplant or during the period of immunosuppression after transplantation. Although T-spot test was recommended to aid in diagnosis of latent TB infection pre- and post-transplant in our centre since 2014, it had not been a routine work yet.

The clinical manifestations of active TB in transplant recipients differ from those in immunocompetent hosts[20–21]. In the present study, the primary symptoms during the first hospital visit of these patients were fever, cough or expectoration, dyspnea, headache, abdominal pain, and superficial lymphadenectasis. Further, 24 (50%) patients had a fever for more than 2 weeks. The CT scan results were also diversified in these patients, showing nodule or speckle, focal proliferation, pleural effusion, hilar or mediastinal lymphadenectasis, or miliary TB. Only 5 (10.4%) patients had classic cavitary changes on chest radiograph. Mathur M[22] also reported that 60% of active TB had cavitation in immunocompetent patients while only 20% in immunocompromised patients.

Moreover, 26 (54.2%) cases had bacteriological confirmation, and the other 22(45.8%) cases were clinically diagnosed without bacteriological confirmation. Among the 34 patients who underwent a T-spot test in the present study, 28 (58.3%) cases were positive and the other 6 cases (12.5%) were T-spot negative. Both T-spot and TST may yield false-negative or indeterminate results in patients receiving high levels of immunosuppressive drugs[23–24]. Therefore, the results should be interpreted with caution.

The rates of disseminated or extrapulmonary TB were higher in post-transplant patients than in immunocompetent hosts as reported[25–26]. About 19 (39.6%) patients in the present study were diagnosed with severe TB, including patients with miliary TB, disseminated TB, or tuberculosis meningitis. However, only about 15% cases were diagnosed with disseminated TB or TB that occurred at extrapulmonary sites in normal hosts.

The atypical symptoms, radiographs, and lab results may cause patient delay or diagnostic delay. In the present study, the mean time from the onset of symptoms to first hospital visit was 24.7 days; in 14 (29.2%) patients, it was more than 30 days. The mean time from a hospital visit to initial treatment was 25.9 days; in 12 (25%) patients, it was more than 30 days. Patient delay or diagnostic delay was reported as a major impediment to effective management of TB in immunocompetent persons in China[27–28] and other countries[29–30]. The treatment delay in patients with TB was found to be associated with increased mortality and clinical severity in immunocompetent patient [30]. However, there was no significant relationship found among diagnostic delay, severity and mortality in our study.

The present analysis of diagnostic delay in patients showed that patients who had fever for more than 2 weeks and who had used antibiotics for more than 2 weeks were significantly associated with TB diagnostic delay more than 30 days. Further data on antibiotic use for the 12 cases of diagnostic delay more than 30 days showed that 11 cases had used antibiotics for more than 2 weeks. Especially seven cases used antibiotics including quinolone, carbapenems, and linenazolid, which were also used as the second-line or add-on agents for rifampicin-resistant and multidrug-resistant TB. The clinical symptoms may be temporarily improved when using the aforementioned antibiotics, but exacerbated or relapsed after discontinuing the use, and finally caused the diagnostic delay.

The attributable mortality of active TB after kidney transplantation in the present study was 18.8%. The analyses of risk factors for death showed those serious complications during anti-TB therapy or in following up period due to TB relapse, such as encephaledema, severe pneumonia, intestinal perforation, and liver function failure, and the following multiple-organ failure, were significantly associated with treatment outcome of death.

This study had several limitations. First, it was a single-center, retrospective, descriptive study with a limited number of cases. It may be the reason why no significant relationship was found for diagnostic delay, severe TB or death. Experience of other transplant centers in China should be considered in future studies if possible. Second, some tests such as T-spot and GenExpert were performed in our hospital not long ago. The experience of quick diagnosis using these new techniques in patients is not sufficient. For example, drug susceptibility testing (DST) results were not available in the initial phase of treatment for some patients in this study. DST may be helpful to objectivate the results in future studies.

In conclusion, the present study indicated that, for post-transplant patients, antibiotics with simultaneous anti-TB effect should be carefully evaluated or avoided if the active TB infection had not been completely excluded. For patients who had a protracted fever for more than 2 weeks or used antibiotics for more than 2 weeks, with no improvement in clinical symptoms or relapse, active TB should be carefully excluded and sometimes diagnostic TB treatment should be considered. Severe complications and the following multiple-organ failure may increase the mortality of TB infection after kidney transplantation. Furthermore, to decrease the morbidity and mortality of active TB after transplantation, more strategies should be adopted to control latent TB pre- and post- transplant[31].

## Supporting information

S1 Table Raw data of subjects in this study. (XLS)

S1 File STROBE checklist. (XLS)

## Abbreviations

AFB: acid-fast bacilli
BCG: bacillus Calmette-Guerin
CRP: C-reactive protein
CSF: cerebrospinal fluid
CT: computed tomography
DTB: disseminated TB
EMB(E): ethambutol
EPTB: extrapulmonary tuberculosis
ESR: erythrocyte sedimentation rate
INH(H): isoniazid
Lfx: levofloxacin
Mfx: moxifloxacin
MRI: magnetic resonance imaging
MTB: Mycobacterium tuberculosis
ND: not done
PPD: purified protein derivative
PTB: pulmonary tuberculosis
PZA(P): pyrazinamide
RFP(R): rifampicin
Rft (L): rifapentine
TB: tuberculosis
TBM: tuberculosis meningitis
TPL: transplantation
TST: tuberculin skin tests
